# Emergence of Edible Plant-Derived Nanovesicles as Functional Food Components and Nanocarriers for Therapeutics Delivery: Potentials in Human Health and Disease

**DOI:** 10.3390/cells11142232

**Published:** 2022-07-18

**Authors:** Sora Q. Kim, Kee-Hong Kim

**Affiliations:** 1 Department of Nutrition Science, Purdue University, West Lafayette, IN 47907, USA; kim2713@purdue.edu; 2 Department of Food Science, Purdue University, West Lafayette, IN 47907, USA

**Keywords:** plant-derived nanovesicles, extracellular vesicles, exosomes, food components, functional foods, phytochemicals, therapy, trans-kingdom interaction

## Abstract

Extracellular vesicles (EVs) are a highly heterogeneous population of membranous particles that are secreted by almost all types of cells across different domains of life, including plants. In recent years, studies on plant-derived nanovesicles (PDNVs) showed that they could modulate metabolic reactions of the recipient cells, affecting (patho)physiology with health benefits in a trans-kingdom manner. In addition to its bioactivity, PDNV has advantages over conventional nanocarriers, making its application promising for therapeutics delivery. Here, we discuss the characteristics of PDNV and highlight up-to-date pre-clinical and clinical evidence, focusing on therapeutic application.

## 1. Introduction

**Figure 1:**
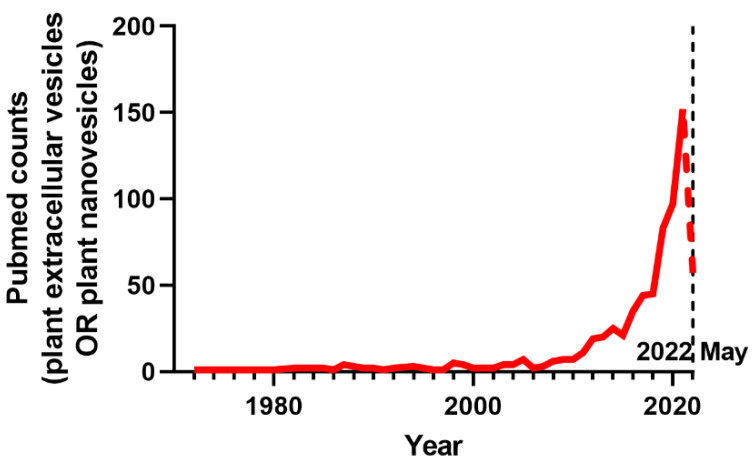
The number of publications per year in PubMed (https://pubmed.ncbi.nlm.nih.gov/ accessed on 1 June 2022) from 1972 to 2021 (as of May 2022). The field of PDNVs has evolved exponentially as shown by the number of publications searched by “plant extracellular vesicles OR plant nanovesicles”.

Consumption of certain foods or their associated components is often linked to health benefits and disease risk reduction. Such bioactive compounds are generally derived from plants: plant-originated micronutrients, sterols, fibers, polyphenols, and other phytochemicals, which scientists have studied to understand their impact on health. Recently, expansion of extracellular vesicle (EV) research (Figure 1) suggested that plant-derived nanovesicles (PDNVs) can be a new member of dietary components with biological impacts.

PDNVs carry a wide array of molecules, including biologically active metabolites, proteins, lipids, and nucleic acids, making them small but mighty vectors capable of modulating metabolic phenotypes in recipient cells. They have gained scientific interest as it became clear that the cell-to-cell communication mediated by the nanoparticle is possible between different species [1,2]. Multiple studies have shown the feasibility of utilizing PDNVs as a means to boost optimal health by complementing insufficient consumption of fruits and vegetables. Furthermore, the nano structure of PDNVs, that allows encapsulation of various types of molecules into the hydrophilic core and the surrounding lipid layer, makes them a natural nanocarrier. In this regard, clinical studies are currently ongoing to determine the effects of PDNVs on human health: whether plant exosomes can deliver curcumin to colon tumors and the normal colon effectively (NCT01294072); whether ginger-derived exosomes, alone or combined with curcumin, ameliorates symptoms of patients with inflammatory bowel disease (NCT04879810); whether grape exosomes the incidence of radiation and chemotherapy-induced oral mucositis (NCT01668849); and whether a natural supplement containing nanovesicles delivered from *Citrus Limon* (L.) juice reduces several cardiovascular risk factors (NCT04698447) [3]. 

Studying PDNVs hold great promise for clinical application, and the field is fast-moving. Therefore, this review aims to discuss the current understanding of PDNVs and to provide up-to-date therapeutic implications and applications by summarizing recent pre-clinical and clinical studies.

## 2. Origin and Nomenclature of PDNVs

According to the guidelines published in 2018 by the International Society for Extracellular Vesicles (MISEV2018), the term “EV” can be used for “particles naturally released from cells that are delimited by a lipid bilayer and cannot replicate” [4]. EVs can be classified based on their subcellular origins: exosomes (less than 150 nm in diameter), microvesicles/microparicles/ectosomes (MVs) (100 nm^−1^ µm in diameter), and apoptotic bodies (1–5 µm in diameter) [5]. Exosomes are the smallest EV subtype generated by the inward budding of an endosomal membrane via the endosomal sorting complex required for transport. The resultant intraluminal vesicles are present inside the multivesicular body (MVB) before being secreted to extracellular space as exosomes when MVB fuses with the plasma membrane. On the other hand, MVs are generally larger than exosomes and originated from direct local outward budding of the plasma membrane. First, lipids and membrane-associated proteins form a cluster in plasma membrane microdomains. In parallel to exosomes, such microdomains recruit soluble components, including membrane proteins, cytosolic proteins, and RNA species [5,6]. This cluster promotes membrane budding and subsequent release. The flipping of phosphatidylserines between the leaflets of the budding membrane is unique to MV biogenesis [6]. Although the terms based on unique biogenesis are generally accepted in the field, specific markers of subcellular origins are not yet established, which often generates inaccurate assignment of EVs [4]. Therefore, physical characteristics, such as size and density, biochemical composition (exosomal protein markers), or descriptions of collecting conditions (e.g., hypoxia, apoptosis, etc.) are recommended as standards to subdivide EVs into groups [4].

Categorizing PDNVs is more challenging and the lack of consensus on acceptable nomenclature is due to confusion generated by obscurities regarding their origins. For instance, the presence of the plasma membrane shed as MVs is not clear in plants. On the other hand, the presence of exosome-like particles in plants has been reported. The fusion of MVBs with the plasma membrane and subsequent release of the small vesicles in the extracellular fluid, apoplast, was first reported in 1967 in a carrot cell culture [7], which is earlier than the observation of exosomes in rat reticulocytes [8]. Since then, plant EVs have been isolated from apoplastic fluid and observed by transmission electron microscopy [9]. In 2017, the evidence of critical roles of plant EVs separated from apoplast in plant defense system was corroborated [10,11], raising the interest to investigate intercellular communication via EVs in plants. Despite accumulating observations of exosomes in plants, it remains unanswered how the nanoparticles can overcome the barrier of the cell wall [12]. Aside from unclear aspects of the biology of plant exosomes, what makes the characterization of the particle more confusing is that only a small portion of studies on PDNVs have appropriately purified particles from apoplastic fluids and thus, few are indeed EVs. Most of the studies have collected particles from fruit/leaf/root juice made by gentle pressing or harsh grinding which would recover not only EVs, but also artificial membranous vesicles as well as nanovesicles which may not be of extracellular origin, such as microsomal fraction [13]. Indeed, Liu et al. directly compared the EVs isolated from apoplastic space with nanovesicles isolated from blending of the model plant *Arabidopsis thaliana* and showed some distinctions between the two types of particles. Although both were similar in size uniformity, membrane charge, and shared some well-known EV proteins, such as annexins, soluble N-ethylmaleimide-sensitive factor attachment protein receptors, and glycosylphosphatidylinositol-anchored proteins, EVs had narrower size range with different density distribution [14]. Furthermore, EVs were more readily taken up by OVCAR5 cancer cells than the leaf-derived nanoparticles, suggesting different fusion efficiency [14]. Despite the blatant error, when considering complicated processes of isolating EVs from apoplast and about 700-fold lower yield than nanovesicles isolated from disruptive blending [14], it is anticipated that future studies will adhere to the general nanovesicle isolation protocol of not distinguishing cellular origin. Thus, the nomenclature should be taken with caution and the term “PDNVs” is used in this review instead of “PDEVs”. 

## 3. Isolation and Characterization Methods of PDNVs

The plant sample collection method varies by studies. Most studies simply indicated that the edible plants they used were purchased from a local market and identified the region. Others described details and took environmental factors into account. For example, Liu et al. described the origin of seeds they obtained and grew their garlic chive in a greenhouse with controlled temperature and light cycle [15]. Furthermore, to keep the maturation levels of leaves constant, they harvested leaves bi-weekly [15]. Perut et al. picked their strawberry samples from their university experimental farm [16]. To ensure the maturation level was comparable across samples, they harvested fully matured strawberries and stored them at −80 °C until analysis [16]. To avoid any confusion and to ensure reproducibility between studies, future work may need to clearly describe farming and harvesting conditions such as climate, region, and degree of maturation. At the same time, it would be interesting to investigate how such conditions alter the quantity and the quality of PDNV production. For instance, Logozzi et al. compared PDNVs grown on organic farms with those in conventional farms and found the former results in greater yield and total anti-oxidant capacity [17].

Pre-processing steps for PDNVs are largely the same with a slight modification depending on the type of fruits or the structure of vegetables. For example, fleshy fruits, such as apple [18,19], blueberry [20,21], orange [22,23], lemon [24], and grapefruit [25,26], having high water content, were crushed/smashed and then homogenized using a blender or squeezed manually or pressed using a juicer. The collected juice was then processed to isolate nanoparticles. Dry fruit, such as nuts, was homogenized with a blender and then mixed with PBS before centrifugation [27]. Similarly, corn was homogenized with distilled water [28]. Oat bran meal was dissolved in PBS and then incubated in a 37 °C water bath for 30 min for supernatant collection and subsequent centrifugation [29]. Root vegetables, such as carrot [30], garlic [31,32,33], ginger [34,35,36,37,38,39,40,41], ginseng [42,43], and turmeric [44], and leafy vegetables including cabbage [45], were prepared similarly to fruits by blending them with or without additional PBS pre- or post-grinding.

Isolation methods of PDNVs follow those established for mammalian EVs as they are considered to be universal tools. Ultracentrifugation (U/C) followed by purification using sucrose-gradient centrifugation is the most common extraction method. Particle size-based isolation methods, such as size-exclusion chromatography (SEC), ultrafiltration (U/F), and tangential flow filtration, are standards as well. Precipitation by polyethylene glycol or commercial isolation kits (e.g., ExoQuick^TM^) is available for extraction. Immunoaffinity is another standard method for capturing mammalian exosomes, but due to high cost, potential exclusion of other subpopulations of nanovesicles, and absence of established surface protein markers and their antibodies for PDNVs, it is not preferred for PDNV isolation [46].

The characterization methods for PDNVs follow established methods for mammalian EVs and are described in other reviews in detail [47,48]. Table 1 shows a concise list of frequently used methods for PDNVs.

## 4. Characteristics of Unmodified PDNVs

### 4.1. Composition/Cargoes

Table 2 lists the studies that investigated the health-promoting effects of PDNVs obtained from edible plants, including various types of fruits and vegetables and summarizes isolation methods, cargoes measured, and experimental model employed. The following section attempts to highlight the cargoes found in PDNVs.

#### 4.1.1. Bioactive Compounds

PDNVs contain a broad range of naturally occurring metabolites, which may contribute to therapeutic effects or health-promoting effects of PDNVs: ginger-derived nanovesicles contain highly enriched 6-gingeraol, 8-gingerol, 10-gingerol, and 6-shogaol compared with ginger slices [34,49] and depletion of the nanovesicles from ginger extracts markedly reduced shogaol detected by thin-layer chromatography method [35]; broccoli-derived nanovesicles contain sulforaphane [50] and HPLC revealed that sulforaphane is more enriched in nanoparticles than in microparticles, while broccoli extracts have little sulforaphane in free form; grapefruit-derived nanovesicles contain naringenin [51]; citrus limon and strawberry-derived nanovesicles contain vitamin C [16]; edible tea flower-derived nanovesicles are enriched in epigallocatechin gallate, epicatechin gallate, epicatechin, vitexin, myricetin-3-*O*-rhamnoside, kaempferol-3-*O*-galactoside, and myricetin; and nanovesicles from citrus limon contain citrate and vitamin C [52]. Furthermore, oat-derived nanovesicles contained a five-times greater percentage of beneficial fiber β-glucan than in oat flour [29] and lemon-derived nanovesicles contained galacturonic acid-enriched pectin-type polysaccharide as the active factor [53], suggesting PDNVs could be developed as nano-size formulated prebiotics. 

Metabolomic analysis of grapefruit revealed distinctly different compositions between nanovesicles and microvesicles isolated from fruit juice. In nanovesicles, organic acids such as glycolic and citric acids were major components, while microvesicles were composed mostly of sugars. Notably, microvesicles contained several anti-cancer, anti-inflammatory compounds, such as myo-inositol, quinic acid, and aucubin [25]. So far, metabolomic data of PDNVs are scarce but it is clear that PDNV compositions would vary not only by plant origin but also by nanovesicle population and isolation methods. The diversity of metabolites needs to be more extensively disclosed to understand their contribution to bioactivity.

#### 4.1.2. Nucleic Acids

Nucleic acids, including small non-coding RNAs, are another component that enables intercellular communications. As shown in Table 2, PDNVs generally contain RNA and particularly a high number of microRNAs (miRNAs). miRNAs regulate a wide array of cellular processes by binding to target mRNAs, leading to either transcript degradation or translation repression [54]. 

PDNVs seem to have selective packaging mechanisms for RNA, which is evidenced by different RNA profiles between plant tissue and the plant-derived nanovesicles. For instance, miRNA cargo is more enriched in ginger-derived nanovesicles, while ginger tissue is rich in tRNAs [55]. Compared with ginger, ginger-derived nanovesicles had 50 differentially expressed miRNAs and 3 of them were more than 4-fold greater in the nanovesicles [41].

As several studies failed to capture increased levels of miRNAs in plasma or tissues after consuming the edible plants that are rich in miRNAs [56,57,58,59], it remains controversial whether the cross-kingdom effects of dietary miRNAs are possible. However, a recent study by Qin et al. showed that plant-derived miRNAs could be protected by co-ingested food components and PDNVs [60], raising the feasibility of utilizing PDNVs as miRNA-mediated therapeutics. Moreover, dried nut-derived nanovesicles were found to contain miR159a and miR156c which could target mammalian *TNFRSF1α* [27]. Indeed, the nut-derived nanovesicles downregulated *TNFα* in the adipocytes and obese mice [27]. Deep sequencing showed that ginger-derived vesicles contain at least 125 different miRNAs [34]. Among them, 124 miRNAs were predicted to regulate the expression of human genes in silico, indicating a potential cross-kingdom regulation of miRNA [34]. 

#### 4.1.3. Lipids

The lipid is another major bioactive component with functionality. Lipids may promote certain cellular responses in recipient cells and facilitate their uptake or containment of cargo materials. For instance, when ginger-derived nanovesicles were treated with heat to denature proteins, or sonicated and treated with RNase to deplete RNAs, the ability to inhibit NLR family pyrin domain containing 3 (NLRP3) inflammasome activity was not affected [38]. In addition, when lipids were extracted from the ginger-derived nanovesicles and reassembled into liposomes, the liposomes retained the ability to suppress NLRP3 inflammasome activity, suggesting nanovesicle lipids were the bioactive molecules [38]. Kumar et al. also showed the bioactivity of lipids from ginger-derived nanovesicles, enhancing Foxa2 expression in intestinal epithelial cells on which RNAs or proteins have no impact [39]. 

PDNVs are high in phospholipid, in part due to the absence of cholesterol which is generally rich in mammalian EVs [61]. PDNVs also contain plant lipids, such as galactolipids [61]. Lipidomic analysis showed that grape-derived nanovesicles are enriched in phosphatidic acids (PA) (53.2%) and phosphatidylethanolamine (PE) (26%) [62]. The whole grape contained a much lower percentage of PA, suggesting selective lipid sorting into the nanovesicles. Ginger-derived nanovesicles also contained PA in a high percentage (~40%) along with plant lipids such as digalactosyldiacylglycerol (DGDG) (30~40%) and monogalactosyldiacylglycerol (MGDG) (~20%) [34,39]. Turmeric, which belongs to the ginger family, also showed high DGDG (~42%) and MGDG (~12%) along with PA (~20%) and phosphatidylcholine (PC) (~16%) [44].

On the other hand, orange juice-derived nanovesicles showed a high percentage of PE (~40%) and PC (~25%) with only ~5% PA [22]. Grapefruit-derived nanovesicles also identified PE and PC as major lipid species (45.52% and 28.53%) [51]. This implies that different fruit families may produce nanovesicles with different lipid compositions, which can explain the high PE/PA ratio in Rutaceae family-derived nanovesicles compared with grape- or ginger-derived vesicles with low PE/PA ratios [22]. 

Regarding plant lipids, an intriguing role of PDNV DGDG in inflammatory signaling was shown by Xu et al. [29]. They showed that DGDG prevented oat nanovesicle β-glucan from interacting with microglial dectin-1 but favored β-glucan binding to hippocalcin. This inhibited the activation of the alcohol-induced brain inflammation signaling pathway.

#### 4.1.4. Proteins

PDNVs contain both cytosolic and membrane proteins. In terms of quantity, approximately 1 mg of orange-derived nanovesicle protein is obtained in 350 mL juice [22]. Presence of the heat shock protein HSP70 or tetraspanins, such as CD9 and CD63, which are generally accepted as exosome markers, were confirmed in PDNVs [26,32]. When the *Citrus*-derived nanovesicles protein dataset was compared with the ExoCarta database, 56.7% proteins overlapped with mammalian exosome proteins [24].

Proteomic analysis is a common approach not only to reveal identifiable markers but also to define the functionality of given samples, and thus, several studies have analyzed the proteome of PDNVs [15,24,51,62,63,64]. The proteome of citrus fruit sac-derived nanovesicles showed the presence of proteins involved in various processes, including glycolysis (e.g., glyceraldehyde-3-phosphate dehydrogenase), glycogenesis (e.g., fructose-bisphosphate aldolase 6), protein folding and transport (e.g., HSP70,HSP80, and PTL39), and cell growth and division (e.g., PTL3 and clathrin-3) [64]. In addition, enzymes such as hydrolases (e.g., ATPases, pectinesterase, phospholipases, amylases, β-galatosidases, and s-adenosylhomocysteine hydrolase) and anti-oxidants (e.g., SODs, CATs, PODs, and GPXs) were identified [64]. Similarly, proteome of other PDNVs also confirmed the presence of various carbohydrates/lipid metabolism-related enzymes [15,24,51,62,63] and clathrin chains and ATPases [15,24,63]. The identification of plasma membrane proteins and membrane-interacting proteins through proteomic analysis confirms the vesicle-like properties of PDNVs.

Despite its usefulness, one of the hindrances to the utilization of proteomics is that the protein sequence database often lacks data for most fruit or vegetables. For instance, a proteomic study on garlic chive-derived nanovesicles used a reference proteome database of *viridiplantae* due to the lack of proteomes information on garlic chive [15]. For the lemon juice-derived nanovesicles, *Citrus* proteomics database was used as *Citrus limon* is a non-model plant species lacking proteomics data [24].

### 4.2. Biodistribution

To delineate the sites of therapeutic effects of PDNVs, it is essential to determine the biodistribution after oral administration or injection. To this end, several studies labeled nanovesicles and tracked the migration and accumulation in vivo. When nanovesicles from Citrus limon were labeled with lipophilic fluorescent tracer DiR (1,10-dioctadecyl-3,3,30,30-tetramethylindotricarbocyanine) and intraperitoneally injected in chronic myeloid leukemia (CML) cells-xenografted NOD/SCID mice, DiR-labeled nanovesicles quickly reached tumor tissue and started to accumulate at 15 min, while the Free-DiR did not get to the tumor site [24], showing effective targeting of the tumor site. Aside from the tumor site, at 24 h post-injection, Free-DiR and DiR-labeled nanovesicles were also found in non-tumor tissues such as the liver, spleen, and partially in the kidneys [24]. In vivo biodistribution study of nanovesicles from tea flowers also showed accumulation in xenograft breast tumor sites, the liver, and the lung after i.v. injection or oral administration [65]. Of note is that fluorescence-labeled nanovesicles were accumulated in the tumor within 24 h of i.v. injection, while it took 48 h for the oral administration, indicating that more nanovesicles may have ended up located in the intestines after oral administration [65]. Nevertheless, oral administration is the most preferred route and is patient-friendly due to non-invasiveness and convenience. When orange juice-derived nanovesicles were labeled with lipophilic dye PKH67 and orally administered to mice, fluorescence was detected in the gut at 6 h post-consumption, implying the main site of action would be the intestine [22]. Similarly, oral gavage of DiR-labeled grapefruit-derived nanovesicles and mulberry bark-derived nanovesicles accumulated at the middle and distal small intestine, cecum, and colon [51,63]. Precisely, the grapefruit-derived nanovesicles were internalized by macrophages in the small and large intestine, Peyer’s patches, mesenteric lymph node, spleen, and liver [51]. The mulberry bark-derived nanovesicles were also found in the macrophages in the spleen but the predominant locations were the gut epithelial cells, Paneth cells, and colon tissue [63]. Intragastric administration of DiR-labeled lemon-derived nanovesicles and PKH26-labeled ginger-derived nanovesicles also resulted in signals located in the gastrointestinal organs [39,66]. Interestingly, PDNVs administered by oral gavage were also found in the brain. Xu et al. showed that oral administration of DiR labeled oat-derived nanovesicles resulted in signal detection in the brain at 1 h post gavage with a gradual decrease after 6 h and preferential uptake by microglial cells in the brain [29]. Similarly, Sundaram et al. showed that garlic-derived nanovesicles given orally traveled to the brain approximately at the same amount as the liver and intestine [33]. Specifically, brain microglial cells but not neuronal cells selectively took the garlic-derived nanovesicles [33]. 

When biodistribution after the i.v. injection and the oral administration were compared using garlic chive-derived nanovesicles with covalent labeling of fluorescence dye, i.v. injection resulted in strong signal detection in the liver, spleen, and kidney, while oral feeding showed strong signals in the GI tract and kidney compared with control [15]. Consistently, when nanovesicles from ginseng were injected intraperitoneally or intravenously, the DiR label was localized in the liver and the spleen, whereas intragastric administration resulted in biodistribution in the stomach and gut [42]. Overall, enteral administration, including oral administration, tends to induce accumulation of the particles in the GI tract, while parenteral administration made through either i.v. or i.p. injection tends to affect mostly the liver, as well as target tissues such as a tumor.

### 4.3. Uptake Mechanism by Recipient Cells

One of the key questions to predict the fate of PDNVs is how they are internalized by recipient cells and how they go through intracellular trafficking. Many studies have effectively demonstrated the incorporation of PDNVs into mammalian cells using imaging methods by labeling nanoparticles with fluorescent dyes (e.g., PKH26) [52], but few studies investigated the uptake mechanism of PDNVs.

One common pathway for the internalization of particles is endocytosis which can be further classified into phagocytosis, clathrin-mediated endocytosis, caveolin-mediated endocytosis, and pinocytosis [67,68]. Based on this, several studies have challenged the uptake of PDNVs using inhibitors specific to each pathway. Apple-derived nanoparticles enter mammalian cells by clathrin-dependent endocytosis [69] as the uptake was inhibited by chlorpromazine and Pitstop 2. On the other hand, caveolae-mediated endocytosis and macropinocytosis inhibitors did not affect the incorporation of the nanovesicles into human colorectal adenocarcinoma Caco-2 cells [69]. In the case of grape-derived nanovesicles, the PDNV uptake was inhibited by macropinocytosis inhibitor, cytochalasin D, as well as bafilomycin A1 and concanamycin A, which are V-ATPase inhibitors in the murine colorectal carcinoma CT26 cell line [62]. The caveolae-mediated endocytosis inhibitor indomethacin and the clathrin-mediated endocytosis inhibitor chlorpromazine did not affect the uptake of PKH26 labeled grape-derived nanovesicles [62]. Ex vivo culture of colon tissues of Lgr5-EGFP-IRES-CreERT2 mice further confirmed the inhibition of the grape-derived nanovesicles uptake by cytochalasin D [62]. On the contrary, uptake of orange-derived nanovesicles was not inhibited by cytochalasin D, but by indomethacin, an inhibitor of caveolae-mediated endocytosis [22]. For grapefruit-derived nanovesicles, the uptake was inhibited by amiloride and chlorpromazine, which are known to inhibit macropinocytosis and clathrin-mediated endocytosis, respectively [51]. Song et al. further demonstrated the potential involvement of surface proteins in the endocytosis. When garlic-derived nanovesicles were incubated in trypsin to remove surface proteins, absorption was inhibited [31]. Garlic-derived nanovesicles are rich in II lectin, a mannose-binding protein. CD98 is highly expressed in different types of cancer and Song et al. showed that CD98 is the primary receptor of garlic-derived nanovesicles via interaction with II lectin [31]. 

The other common pathway for the internalization of particles is non-specific uptake [67], which occurs through hydrophobic or electrostatic interactions. Parolini et al. showed direct fusion of the exosome with the plasma membrane of melanoma cells, which was facilitated under acidic conditions than in buffered conditions [70]. Furthermore, the proton pump inhibitor reduced the level of exosome entry into cells [70]. Based on these findings, it is conceivable that oral administration of PDNVs and subsequent absorption may be affected by changes in pH of the gastrointestinal tract. Indeed, in vitro setting mimicking digestion conditions showed PBS and intestine-like solution resulted in a slight negative charge in PDNVs and a slight positive charge in PDNVs in acid solution [34,44]. Currently, the absence of evidence leaves this open to discussion. 

Collectively, mechanisms of uptake of PDNVs into mammalian cells may vary by plant species as well as recipient cell types. However, most PDNV experiments utilized certain sets of inhibitors to block specific pathways and other potential uptake mechanisms were rarely tested. Sundaram et al., for example, showed that PA (36:4) in the surface of the garlic-derived nanovesicles interacted with BASP1 protein in the brain for efficient particle uptake and BASP1 knockdown in BV2 brain microglial cells resulted in decreased uptake level [33]. This suggests that lipids composition of the PDNV membrane may modulate the selectivity and efficiency of PDNV uptake. Therefore, a future study needs to investigate the role of microenvironments, specific proteins, and specific lipid species of the PDNV membrane on PDNV uptake. The potential contribution of direct fusion rather than endocytosis to PDNV uptake also needs to be tested. At this point, due to lack of information of PDNV uptake mechanisms, we can only speculate that PDNV-mediated cargo transfer to mammalian cells is likely to occur by several different mechanisms depending on the biological contexts rather than a single bona fide mechanism.

**Table 2:** Health benefits of natural PDNVs.

Origin	Isolation	Contents Identified	Size ^1^ (Measurement Method)	Health Effects	Model	References
Aloe vera	U/C and tangential flow filtration	-	50–200	Anti-oxidative effects	Human keratinocytes (HaCaT)	[71]
Apple	-		-	Intestinal function	Human colon epithelial cell line (Caco-2)	[18]
	U/C	-	152 ± 32.3 (tRPS)	Anti-inflammation	Monocyte cell line (THP-1), fibroblast cell line (NCTC L929)	[19]
Blueberry	U/C	Cyanidin-3-*O*-glucoside	198 ± 112 (DLS)	Anti-inflammation	Human endothelial cell line (EA.hy926)	[20]
	Filtration (1 µm), incubation with 8% PEG8000 and 10,000× *g* centrifugation	Protein, RNA, lipid	189.62 (DLS)	Anti-oxidative effects	Human hepatocarcinoma cell line (HepG2), HFD-fed C57BL/6 mice	[21]
Bitter melon	Electrophoresis and dialysis	Protein, RNA, microRNAs	100–300 (NTA)	Anti-inflammation, anti-cancer and synergism with 5-FU	Human oral squamous cell carcinoma cell line (CAL 27, WSU-HN6)	[72]
Broccoli	U/C	Sulforaphane	32.4 (DLS)	Protection against DSS-induced colitis	DSS-induced colitis in C57BL/6 mice	[50]
Butterbur (*Petasites japonicus*)	U/C	-	122.6 (DLS)	Immunomodulation	Bone marrow-derived dendritic cells (BMDC)	[73]
Cabbage, red cabbage	SEC, U/F	-	~100 (TEM)	Anti-inflammation and anti-apoptosis	HaCaT, human dermal fibroblasts (HDF), mouse macrophage cell line (RAW264.7)	[45]
Carrot	U/F and SEC	-	143.9 (NTA)	Anti-oxidative effects	Embryonic rat heart-derived cardiomyoblasts (H9C2), neuroblastoma cells (SH-SY5Y)	[30]
Corn	U/C	-	80 (DLS)	Anti-cancer (colon cancer)	Mouse colon cancer cell line (Colon26), xenograft model	[28]
Garlic	U/C	Proteins	<150 (TEM)	Anti-inflammation	HepG2	[31]
	Aqueous two-phase systems	Proteins	50–150 (NTA)	Anti-cancer	Human kidney carcinoma (A498), human lung carcinoma (A549), HDF	[32]
	U/C and sucrose gradient centrifugation	Lipids, proteins, miRNAs	~200 (NTA)	Anti-obesity	Primary neuronal cell culture, wildtype C57BL/6, IDO1^−/−^ and AHR^−/−^ male mice with diet-induced obesity	[33]
Garlic chive	U/C and sucrose gradient centrifugation	Lipids, proteins, and RNAs	113–153 (NTA)	Anti-inflammation	Acute liver injury model by administration of D-galactosamine and LPS, C57BL/6 with dietinduced obesity	[15]
Ginger	U/C and sucrose gradient centrifugation	6-gingerol and 6-shogaol, miRNAs	~230 (DLS)	Protection against DSS-induced colitis	RAW 264.7, Caco-2BBE, Colon-26, DSS-induced colitis in FVB/NJ mice, IL10^−/−^ C57BL/6 mice	[34]
	sucrose gradient centrifugation	Lipids, shogaols	386.6 (DLS)	Protection against alcohol-induced liver damage	Wildtype, MyD88 KO, TRIF KO, and TLR4 KO C57BL/6J	[35]
	polyethylene glycol-based method	SARS-CoV-2 targeting miRNAs	-	Treatment of SARS-CoV-2	in silico analysis predicting SARS-CoV-2 targeting miRNAs	[74]
	U/C and sucrose gradient centrifugation	miRNA	~180 (DLS)	Treatment of SARS-CoV-2	A549 cells; C57BL/6 mice intratracheally injected with exosomes^Nsp12Nsp13^	[55]
	U/C and sucrose gradient centrifugation	Phosphatidic acid, miRNAs	~200 (NTA)	Treatment of periodontitis	periodontitis mouse model, *Porphyromonas gingivalis*	[36]
	U/C and sucrose gradient centrifugation	miRNAs, lipids	206.8 (NTA)	Modulation of gut bacteria	C57BL/6 mice, human subjects	[37]
	U/C	RNAs, protein, lipids	132 (NTA)	Anti-inflammation	Primary BMDMs	[38]
	Centrifugation (10,000× *g*, 1 h) and sucrose gradient centrifugation	Proteins, lipids	250 ± 72 (NTA)	Anti-obesity	Murine colon adenocarcinoma cell line (MC-38) and Caco-2 cells, C57BL/6 mice	[39]
	Centrifugation (10,000× *g*, 1 h) and sucrose gradient centrifugation	-	-	Prevention of insulin resistance caused by obesity	C57BL/6 male mice with diet-induced obesity	[40]
	Commercial exosome isolation kits	miRNAs	156 ± 36 (DLS)	Anti-inflammation	Caco-2 cells	[41]
Ginseng	U/C and sucrose gradient centrifugation	Amino acids, nucleotides, lipids/fatty acids, organic acids	344.8 (DLS)	Inhibition of melanoma growth and M2-like polarization of macrophage	Murine melanoma cell line (B16F10) xenograft in C57BL/6 mice, BMDMs	[42]
	U/C	Lipids	92.04 ± 4.85 (DLS)	Anti-senescence and anti-pigmentation	Neonatal human epidermal keratinocytes (HEK), HDF cells, human epidermal melanocytes (HEM)	[43]
	U/C and sucrose gradient centrifugation	-	-	Improvement of PD-1 mAb therapy in cancers	B16F10 cells, mice bearing CT26 tumor, mouse breast cancer cell line (4T1)	[75]
Grape	sucrose gradient centrifugation	-	91.28–712.4 (DLS)	Potential induction of Lgr5+ intestinal stem cells	Male Sprague-Dawley rats	[76]
	sucrose gradient centrifugation	Protein, lipid, miRNA	380.5 ± 37.47 (DLS)	Protection against DSS-induced colitis and modulation of intestinal tissue renewal	C57BL/6 mice, Lgr5-EGFP-IRES-CreERT2 mice, ex vivo crypt culture, CT26 cells	[62]
Grapefruit	Polyethylene glycol-based method	SARS-CoV-2 targeting miRNAs	-	Treatment of SARS-CoV-2	in silico analysis predicting SARS-CoV-2 targeting miRNAs	[74]
	U/C	Organic acids, amino acids, others	-	Anti-cancer	Human Melanoma Cell Line (A375)	[25]
	aqueous two-phase systems	Proteins	~82–~239(NTA)	Wound healing	HaCaT, human vascular endothelial cells (HUVEC)	[26]
Lemon	U/C	Proteins	50–70 (EM)	Anti-cancer	A549, human colorectal adenocarcinoma cell line (SW480), human CML cell line (LAMA84), CML xenograft model	[24]
	U/C	Citrate, vitamin C, short RNAs	30–100 (TEM)	Anti-oxidant activity and type I collogen synthesis	Mesenchymal stromal cells (MSC)	[52]
	citraVes™	Sugars (glucose, fructose, sucrose), organic acids (isocitric, malic acid), flavonoids (eriocitrin, hesperidin)	40–100 (DLS)	Reduction of waist-circumference and LDL level	Healthy volunteers	[3]
	U/C or electrophoresis and dialysis	-	~100 (NTA)	Anticancer	gastric cancer cell lines (AGS, BGC-823, SGC-7901 cells), xenograft mice model	[66]
	Sucrose gradient centrifugation	-	-	Improvement of probiotic function	*Clostridioides difficile*-infected mice	[77]
	Sucrose gradient centrifugation	Polysaccharides	~180.5 (DLS)	Improvement of probiotic function	C57BL/6 mice, *Lactobacillus rhamnosus GG* culture	[53]
	U/C	Citrate	~100 (NTA)	Anti-cancer	Human colorectal cancer cell lines (HCT116 wildtype, HCT116 p53^−/−^, HCT-15, SW480 cells)	[78]
Mulberry bark	U/C	Protein, lipid, RNAs	151.3 ± 45.4 (NTA)	Protection against DSS-induced colitis	DSS-induced colitis in C57BL/6 mice, gut epithelium-specific knockout mice of COPS8	[63]
Nut	U/C	Lipids, miRNAs	100–500 (DLS)	Anti-inflammation	3T3 adipocytes, in vivo adipose tissue	[27]
Oat	U/C and Optiprep gradient purification	Proteins, lipids, polysaccharides	~135 (NTA)	Anti-inflammation	C57BL/6 mice with 5% ethanol liquid diet to induce alcohol induced brain inflammation	[29]
Orange	U/C, SEC	Lipids, carbohydrates, amino acids, organic acids, alcohol	60–140 (NTA)	Treatment of obesity-associated intestinal complications	In vitro intestinal barrier model (Caco-2+HT-29-MTX), HFHSD mice	[22]
	U/F and U/C	-	~62, ~247 (DLS)	Anti-inflammation	Caco-2	[23]
Sap from plants	Filtration and centrifugation	-	50–200 (NTA)	Anti-cancer	Human squamous carcinoma cells (A431), human breast carcinoma cells (MCF7), human breast carcinoma cells (MDA-MB-231), murine melanoma cells (B16BL6, B16F1)	[79]
Shiitake mushroom ^2^	U/C	RNA, proteins, lipids	~115 (NTA)	Anti-inflammation and protection against acute liver injury	Primary BMDMs, C57BL/6 J mice	[80]
Strawberry	U/C	Vitamin C	30–191 (TEM)	Anti-oxidative effects	Adipose-derived mesenchymal stem cells (ADMSCs)	[16]
Tartary buckwheat	U/C	miRNA, proteins	141.8 (NTA)	Modulation of gut bacteria	*Escherichia coli* and *Lactobacillus rhamnosus* culture, human fecal sample	[81]
Tea flowers	U/C and sucrose gradient centrifugation	EGCG, epicatechin gallate, epicatechin, vitexin, myricetin-3-*O*-rhamnoside, kaempferol-3-*O*-galactoside, myricetin, proteins, lipids	131 (DLS)	Anti-cancer	MCF-7, 4T1, A549, Human cervical carcinoma (HeLa)	[65]
Turmeric	U/C and sucrose gradient centrifugation	Lipids, proteins, curcumin	177.9 (DLS)	Protection against DSS-induced colitis	Colon-26, Caco-2BBE, RAW 264.7 cells, DSS-induced colitis in FVB/NJ female mice, NFκB-RE-Luc transgenic female mice	[44]
Wheat grass	Commercial exosome isolation kits	-	40–100 (DLS)	Regeneration	HDF, HUVEC, HaCaT cells	[82]

^1^ Size is expressed as either a range or an average. ^2^ A mushroom is not a plant but due to its nutritional similarity with vegetables, the shiitake mushroom was included in this review.

## 5. Health Benefits of Unmodified PDNVs

As Table 2 shows, PDNVs are involved in a variety of cellular processes and impact our health. In this section, we highlighted key health benefits among them.

### 5.1. Anti-Cancer Activity of PDNVs

Anti-tumor effects of PDNVs have been tested in multiple cancer types and they consistently showed anti-proliferative effects that are greater in cancer cells than in non-cancerous cells: citrus limon-derived nanovesicles attenuated cell growth of tumor cells including A549, SW480, and LAMA84 but had no effects in HS5, HUVEC, and PBMC cells [24]; vesicles from lemon, grapefruit, and sweet and bitter oranges’ juice inhibited cell viability of MCF7, A549, and A375 but not human keratinocytes, HaCat cells [25]; nanovesicles from *Dendropanax morbifera* and *Pinus densiflora* sap suppressed cell viability in MDA-MB-231, MCF7, and A431 but not as much in MCF10A and human normal fibroblast cells [79]; garlic-derived nanovesicles caused apoptotic cell death in A498 and A549 carcinoma while HDF cells were unaffected [32]; edible tea flower-derived nanovesicles inhibited MCF-7, 4T1, A549, and HeLa cells but not HUVEC cells and HEK293T cells [65]; corn-derived nanovesicles inhibited colon26 cell growth but not NIH3T3 or RAW264.7 cells [28].

Different mechanisms were involved in the anti-cancer effects of PDNVs. Ginseng- and corn-derived nanovesicles activated immune cells and increased pro-inflammatory cytokines, inducing cancer cell death [28,42]. The generation of reactive oxygen species (ROS) is another key reaction that leads to the anti-cancer effects. Lemon-derived nanovesicles generated ROS which upregulated GADD45α, mediating cell cycle arrest and apoptosis in gastric cancer cells [66]. Nanovesicles from tea flowers containing bioactive compounds such as polyphenols and flavonoids also generated ROS, resulting in cancer cell inhibition [65]. Meanwhile, vascular endothelial growth factor (VEGF) mediates angiogenesis in cancer, which is key for tumor development and growth. Garlic-derived nanovesicles and lemon-derived nanovesicles suppressed angiogenic VEGF, showing the inhibitory role of PDNVs in vascularization [24,32].

### 5.2. Modulation of Metabolic Diseases

Obesity is a global epidemic and is accompanied by low-grade systemic inflammation and other comorbidities, including diabetes, hypertension, heart disease, hypertension, and dyslipidemia. Several studies suggest PDNVs reverse the complications induced by obesity. When garlic-derived nanovesicles were orally administered to high-fat diet (HFD) fed mice, plasma inflammatory cytokines (e.g., IL-1β, IL-6, IL-17A, IFN-γ, and TNF-α) decreased to the levels comparable to those of lean mice [33]. Furthermore, a 6-week, daily treatment induced significant weight loss (~20 g) with an improved plasma lipid profile and insulin sensitivity, and decreased hepatic fat depots and brain inflammation [33]. When ginger-derived nanovesicles were administered to HFD-fed mice in the drinking water at a concentration of 6 × 10^8^/mL for 12 months, the mice that received nanovesicles were resistant to HFD-induced weight gain, liver weight gain, intestinal dysfunction, insulin resistance, and plasma inflammatory cytokines with longer lifespan compared to those in the vehicle control group [39]. On the other hand, a 4-week treatment of orange-derived nanovesicles in high fat high sucrose diet (HFHSD) fed mice failed to reduce body weight and did not restore insulin sensitivity or glucose tolerance [22]. However, the jejunum, where the orange-derived nanovesicles accumulated, showed increased villi size and increased gene expressions related to tight junctions such as *CLDN1*, *OCLN*, and *ZO1*, suggesting that these nanovesicles could promote recovery of intestinal functions disrupted by HFHSD [22]. Preliminary results from a 12-week intervention in healthy subjects suggested cardiometabolic health benefits of lemon-derived nanovesicle consumption [3]. When the normolipidemic subjects were given the natural supplements, CitraVes^TM^, their waist circumference and LDL level decreased [3]. Future studies need to confirm the beneficial effects of the lemon-derived nanovesicles in subjects with CVD or at high risk.

### 5.3. Anti-Oxidative Effects of PDNVs

Plants synthesize various non-enzymatic anti-oxidants, including ascorbic acid, glutathione, α-tocopherol, flavonoids, carotenoids, proline, and phenolic acids, as well as enzymatic anti-oxidant defense systems, such as superoxide dismutase, catalase, glutathione peroxidase, and glutathione reductases, to reduce toxicity imposed by ROS [83,84]. In line with this, several studies confirmed the anti-oxidant effects of PDNVs that resemble their plant origin. Nanovesicles from citrus limon and strawberry rescued human MSC from H_2_O_2_-induced oxidative stress, which was in part due to vitamin C found in the nanovesicles (0.009 nM vitamin C/µg and 0.416 nM vitamin C/µg, respectively) [16,52]. Nanovesicles obtained from aloe vera peels also reduced ROS levels in H_2_O_2_-treated HaCaT cells and displayed anti-oxidant activity with upregulation of *NRF2* gene, a key transcription factor regulating cellular anti-oxidant response, expression together and its downstream genes *HO-1*, *Catalase*, and *SOD* [71]. Similarly, carrot-derived nanovesicles inhibited ROS generation and apoptosis in cardiomyoblasts and neuroblastoma cells by upregulating *Nrf2*, *HO-1*, and *NQO-1* gene expressions compared with H_2_O_2_-treated control cells [30]. Further research is warranted to determine the anti-oxidant effects of PDNVs in vivo.

### 5.4. Anti-Inflammatory Effects of PDNVs

PDNVs derived from various edible plants, such as garlic chive [15], shiitake mushroom [80], bitter melon [72], broccoli [50], dried nuts [27], and others, exhibited anti-inflammatory properties (Table 2). Nanovesicles from garlic chive and shiitake mushroom significantly inhibited NLRP3 inflammation when stimulated by lipopolysaccharide (LPS) and palmitate in vitro in bone marrow-derived macrophages (BMDMs) [15,80]. In the disease context, the garlic chive and shiitake mushroom nanovesicles alleviated inflammation in acute liver injury in mice that were generated by administrating D-galactosamine and LPS [15,80]. The anti-inflammatory potential of garlic chive nanovesicles was further validated in epididymal white adipose tissue in obese mice, which represent chronic inflammation states, and showed downregulation of inflammatory cytokine expressions [15]. Yang et al. showed bitter melon-derived nanovesicles contained selective microRNAs, 11 of which are predicted to regulate the expression of NLRP3 mRNA [72]. 

Colitis is a chronic inflammation of the inner lining of the colon. Since PDNVs after oral administration are located predominantly in the GI tract and can have biological action on the site, their therapeutic effects on colitis were investigated [34,50]. When broccoli-derived nanovesicles carrying sulforaphane were administered orally, they activated adenosine monophosphate-activated protein kinase in dendritic cells and prevented dextran sulfate sodium (DSS)-induced colitis [50]. Another study showed oral administration of ginger-derived nanoparticles downregulated the pro-inflammatory cytokines (*TNF-α*, *IL-6* and *IL-1β*), and upregulated anti-inflammatory cytokines (*IL-10* and *IL-22*) in the DSS-induced colitis model [34].

Among many inflammatory markers, TNF-α is a key cytokine tightly connected to the impairment of glucose tolerance and insulin sensitivity. Aquilano et al. showed that nanovesicles isolated from dried nuts attenuated the TNF-α signaling pathway in adipocytes and improved glucose tolerance as well as inflammatory cytokine profile in visceral adipose tissue in a diet-induced obesity model [27]. Furthermore, they found plant miR159a and miR156c in dried nuts were responsible for the changes as they have high complementarity with the mammalian Tnfrsf1a transcript [27]. Another study also showed that blueberry-derived nanovesicles counteracted the response to TNF-α in human vascular endothelial cells [20]. 

In summary, these findings suggest that PDNVs isolated from certain edible plants can be developed to prevent acute and chronic inflammatory diseases. Thus, it would be interesting to test their effects on other inflammatory diseases such as arthritis, bronchitis, or hepatitis. 

### 5.5. Modulation of Pathogenic/Probiotic Bacteria

PDNVs are known to modulate pathogenic or gut bacteria functions. For example, ginger-derived nanovesicles interact with hemin-binding protein 35 on the surface of the periodontal pathogen *Porphyromonas gingivalis* and reduce its pathogenicity [36]. Specifically, PA in the nanovesicle membrane and miRNAs inside were responsible for the reduction of FimA expression and prevention of attachment of the bacteria to oral epithelial cells [36]. Lemon-derived nanovesicles are another example that inhibits the pathogen *Clostridioides difficile*, which causes diarrhea and pseudomembranous colitis by enhancing the survivability of probiotics (*Streptococcus thermophilus* ST-21 and *Lactobacillus rhamnosus* GG (LGG)). The two probiotics synergistically worked, resulting in increased aryl hydrocarbon receptor (AhR) ligand production such as indole-3-lactic acid, indole-3-carboxaldehyde (I3A), and lactic acid [77]. The AhR signaling pathway enhances intestinal IL-22 production and maintains the levels of mucosal anti-microbials and, thus, intestinal barrier function. Teng et al. used AhR knockout mice, IL-22 knockout mice, and germ-free mice to thoroughly show that ginger-derived nanovesicles were taken up by probiotic LGG and induce I3A, which promoted activation of the AhR pathway and IL-22 production [37].

With regards to gut-related disease settings, mulberry bark-derived nanovesicles showed protective effects against DSS-induced colitis by inducing various anti-microbial peptides and reducing harmful bacteria [63]. Specifically, the nanovesicles promoted heat shock protein family A (HSP70) member 8-mediated activation of the AhR signaling pathway in intestinal epithelial cells. This led to the induction of COP9 constitutive photomorphogenic homolog subunit 8 (COPS8), which induced the anti-microbial peptide [63]. Meanwhile, Lei et al. showed that lemon-derived nanovesicles could enhance the resistance of *Lactobacillus rhamnosus* GG to bile. Bile creates harsh environments for bacteria to survive in the small intestine and the lemon-derived nanovesicles were able to downregulate bacterial Msp1 and Msp3 proteins, resulting in decreased bile accessibility to the cell membrane [53].

Overall, different PDNVs are anticipated to have different biological impacts on microbiota and further investigations are expected to broaden our knowledge.

### 5.6. Treatment of SARS-CoV-2

Due to the pandemic outbreak of COVID-19, there are ongoing efforts to develop anti-viral therapeutic agents. The viral genome codes for the structural protein of SARS-CoV-2, including spike (S), envelope (E), matrix (M), and nucleocapsid (N), as well as non-structural proteins RNA-dependent RNA polymerase (nsp12), helicase (nsp13), mRNA capping (nsp14 and nsp16), and fidelity control (nsp14) [85]. While our understanding of the role of PDNVs in SARS-CoV-2 transmission and pathogenesis is still in its infancy, Teng et al. showed that ginger-derived nanovesicle miRNAs have sequences that bind to SARS-CoV-2 genes. The rlcv-miR-rL1-28-3p inhibited SARS-CoV-2 S gene and alymiR396a-5p inhibited Nsp12 gene in A549 cells [55]. Similarly, Kalarikkal et al. analyzed PDNV-derived miRNAs in silico and showed that a selection of miRNAs might target the SARS-CoV-2 transcriptome, whose presence was validated in the grapefruit and ginger [74]. These studies suggest the potential use of PDNVs in alleviating SARS-CoV-2-related outbreaks, although more pre-clinical and clinical studies need to be performed.

## 6. PDNVs as a Nanocarrier

For administrated bioactive molecules to exert their biological effects as intended, they must endure an unfavorable physiological environment and reach their target site in a proper amount at the right time. A wide range of drug delivery system techniques has been devised to enhance the efficacy of therapeutic agents, including nanocarriers. Organic, inorganic, metallic, and polymeric nanostructures such as liposomes, solid lipid nanoparticles, dendrimers, and micelles are examples of nanomaterials on the market or under pre-clinical investigation [86]. Nanocarriers offer several advantages over conventional forms of drugs in that cargoes are protected from unwanted degradation and show improved retention and penetration to tissue. However, application to clinical settings is impeded by several issues such as cytotoxicity [87], ecotoxicity [88,89], and mass production at affordable prices. For these reasons, naturally occurring EVs emerged as an alternative to artificially synthesized vehicles. EVs can cross natural barriers such as the blood-brain barrier [90], which is an ideal trait for carrier molecules. Second, EVs can circulate in the system for a relatively long term as they are encapsulated and protected from enzymatic degradation by the lipid bilayer structure [91]. Thus, cargoes stay stable in circulation. Lastly, they can be immunological because they are from biocompatible cells, making them less likely to trigger immune responses [92]. When compared with mammalian cell-derived EVs, PDNVs could offer more advantages because many mammalian, cell-derived EVs are engaged in tumor biology, and their potential biohazards are not clearly identified yet. In addition, mammalian cell cultures require animal components, including fetal bovine serum, which can cause critical safety problems in clinical applications [93]. Furthermore, PDNVs are more cost-effective than mammalian, cell-derived EVs. For instance, the average yield of cabbage-, red cabbage-, and carrot-derived nanovesicles were 1.504 × 10^11^ particles/g, 1.098 × 10^11^ particles/g, and 3.24 × 10^11^ particles/g each [30,45], while the retail prices of cabbage, red cabbage, and carrot reported by the United States Department of Agriculture in 2016 are only $0.001367/g, $0.002249/g [45], and $0.001698/g, respectively. Vesicles could be obtained as a by-product of crops, such as roots or leaves, which are otherwise non-profitable, providing profits. For instance, nanovesicles obtained from tomato root exudates without infections harbored similar proteins typically present in plant apoplastic vesicles and exerted anti-fungal activity in vitro [94]. In terms of stability, grapefruit-derived nanovesicles were resistant to in vitro digestion by gastric pepsin and pancreatic and bile extract solution [51]. Ginger-derived nanovesicles were also tested for stability by incubating them in a stomach-like solution (pepsin solution in pH 2.0) or first in the stomach-like solution and then in small intestine-like solution (bile extract and pancreatin solution with pH adjusted to 6.5) [34]. The results showed the ginger-derived nanovesicles were stable in those solutions with a slight reduction in size and zeta potential changed according to the surrounding pH: negative charge in the PBS and intestine-like solution; and slightly positively charged in the stomach-like solution [34]. Turmeric-derived nanovesicles also maintained nano-scale size under different pH solutions with their size increased, undergoing similar zeta potential changes as ginger-derived nanovesicles [44]. These collectively suggest PDNVs would survive a harsh environment in the GI tract when orally consumed in food form or purified form due to their membrane versatility.

## 7. Modification of PDNVs to Better Serve as a Nanocarrier

PDNVs can undergo additional processes to incorporate therapeutic cargoes into the interior. The loading methods include co-incubation, electroporation, sonication, chemical transfection, freeze-thaw method, and extrusion [95,96]. Table 3 shows a list of studies that utilized PDNVs as nanocarriers. Co-incubation is helpful for the cargoes that can diffused into the interior of the vesicles through the membrane and is relatively simple, but its loading efficiency is low. Incubation of cabbage-derived nanovesicles with miRNA and transfection reagent or incubation of cherry-derived nanovesicles with miRNA on ice successfully incorporated miRNA into the interior [45,97].

The surface of PDNVs could also be modified to achieve targeted delivery, increased stability, and efficient uptake. One example of nanoparticle surface modification is polyethylene glycol coating (PEGylation), which decreases immunogenicity and increases systemic circulation time [98]. When nanovesicles from *Asparagus cochinchinensis* were PEGylated, vesicles were retained in circulation for a prolonged time and accumulated more in tumor tissue without side effects. Patching of heparin-cRGD (tripeptide Arg-Gly-Asp motif) peptide conjugates onto lemon-derived nanovesicles [99] or patching of doxorubicin loaded heparin-cRGD- based nanoparticles onto the surface of grapefruit-derived nanovesicles [100] are another examples of surface modification. Since RGD motifs are recognized by αvβ3 integrins in proliferating endothelium of tumors [101], such surface modification could improve the tumor-targeting capacity of PDNVs. Moreover, heparin helps to increase the stability and in vivo retention time due to its anti-complement activation capacity [102]. 

Chen et al. [103] used tris (2-carboxyethyl) phosphine (TCEP) to selectively reduce disulfides on proteins of grape and ginger-derived nanovesicles. TCEP is a mild reducing agent that does not react to other molecules, such as phospholipids, and thereby, membrane integrity is preserved. The TCEP-reduced nanovesicles were then reacted with maleimide derivatized transferrin, a cancer-targeting ligand, increasing the target-specificity of EVs [103].

**Table 3:** PDNVs as nanocarriers.

Origin	Isolation	Modification	Loading Method	Cargoes	Administration Method	Therapeutic Potential	References
Acerola cherry	Commercial exosome isolation kits	Cargo loading	incubation	miRNA	Oral administration	Nucleic acid delivery to the digestive tract	[97]
Aloe	U/C	Cargo loading	incubation	Indocyanine green	Intra-tumoral injection	Selective over-heating tumor cells with infra-red; transdermal property (potential development as a non-invasive agent for skin cancer)	[104]
*Asparagus cochinchinensis*	Sucrose gradient U/C	Surface (PEGylation)	-	(inherent) lipids, proteins, and RNAs	i.v. injection	Anti-cancer (hepatocarcinoma)	[105]
Cabbage, red cabbage	SEC, U/F	Cargo loading	Incubation with miRNA and transfection reagent; incubation with high concentration drug	miR-184; doxorubicin	Cell culture media	Nucleic acid or chemotherapeutic drug delivery	[45]
Grapefruit	Sucrose gradient, U/C	Cargo loading	Conjugation	Methotrexate (MTX)	Oral administration	Anti-colitis	[51]
	-	Surface (patching doxorubicin loaded heparin/cRGD-based nanoparticles (DNs) onto the surface of grapefruit EVs)	-	-	i.v. injection	Anti-cancer (glioma)	[100]
Lemon	U/C and sucrose gradient	Surface (patching heparin-cRGD)	Incubation with moderate stirring	Doxorubicin	i.p. injection	Anti-cancer (ovarian cancer) and overcoming multidrug resistance	[99]

## 8. Plant-Derived Lipid Reassembled Particles

In order to increase the uniformity and reproducibility of nanoparticles, some studies used lipid extracts of PDNVs and reconstructed new lipid nanocarriers (Table 4). Caveats of this recombinant method are increased complexity of nanocarrier preparation procedures and potential loss of inherently contained cargoes, and thus losing key properties of unmodified PDNVs. Despite these disadvantages, Wang et al. showed that reassembled grapefruit-derived nanoparticles (GNPs) could be used to deliver chemotherapeutic agents (JSI-124, paclitaxel) and siRNAs to cancer cells, such as brain tumor cells (GL26) and colon cancer cells (CT26, SW620) [106]. In other studies, GNPs were further modified by the leukocyte plasma membrane coating or folic acid coating to further confer a desirable multilayer surface [107,108]. Indeed, folate coating enhanced targeted delivery to folate receptor-positive brain tumor tissue and leukocyte plasma membrane coating augmented delivery specificity to inflammatory tissue [107,108]. 

A ginger-derived nanovector loaded with RNAs was produced by ginger-derived nanovesicle lipid extraction and suspension with RNAs, followed by UV irradiation and sonication [55]. TEM analysis showed reconstituted nanovectors and parental nanovesicles were similar morphologically and FACS analysis showed the nanovector is taken up by both h F4/80+ macrophages and EpCAM+ lung epithelial cells after intratracheal injection [55], showing that the reconstituted nanovector retains its ability to be efficiently taken up. In terms of transfection efficiency, the nanovector loaded with aly-miR396a-5p delivers the miRNA more efficiently than the parental, ginger-derived nanovesicle or polyethylenimine and less than RNAiMAX in A549 cells. The superior delivery efficiency of the miRNA compared with gold nanoparticles after intratracheal injection again supports the promising application of the ginger-derived nanovector [55]. Furthermore, when the effect of lipids was tested by manipulating the level of predominant lipids PA, PC, or PE in the ginger-derived nanovector, additional PE in the nanovector resulted in increased uptake by A549 cells, whereas PA and PC inhibited uptake [55]. Other studies that used a ginger lipid-derived nanovector also confirmed the superior ability of particles to liposome in terms of biocompatibility and efficient delivery of cargoes such as siRNA and chemotherapeutic agent doxorubicin [109,110].

**Table 4:** Nanoparticles made of plant-derived lipids.

Origin	Isolation and Modification	Cargoes Loaded	Administration	Therapeutic Potential	References
Grapefruit	Sucrose gradient, U/C; reassembled	Chemotherapeutic agents (JSI-124, Paclitaxel), luciferase gene siRNA	i.v. injection	Targeted delivery of chemotherapy drug to colon tumors in xenograft model (CT26 and SW620 colon cancer in SCID mouse)	[106]
	Sucrose gradient U/C; reassembled and folic acid coated	miRNA	Intranasal administration	Targeted delivery to folate receptor-positive brain tumor	[107]
	Sucrose gradient U/C; reassembled and leukocyte plasma membrane-coated	Doxorubicin, curcumin	i.v. injection	Targeted delivery to inflammatory tumor tissue	[108]
Ginger	Reassembled	siRNA	Oral administration	Treatment of ulcerative colitis	[109]
	Reassembled	miRNA	Oral administration	Protection against DSS-induced colitis	[37]
	Reassembled and folic acid-coated	Doxorubicin	i.v. injection	Treatment of colon cancer	[110]
	Reassembled (ultrasonication)	miRNAs	Intratracheal injection	Inhibitory effect on the expression of inflammatory cytokines and viral Nsp12 and S expression.	[55]

## 9. Challenges and Opportunities

**Figure 2:**
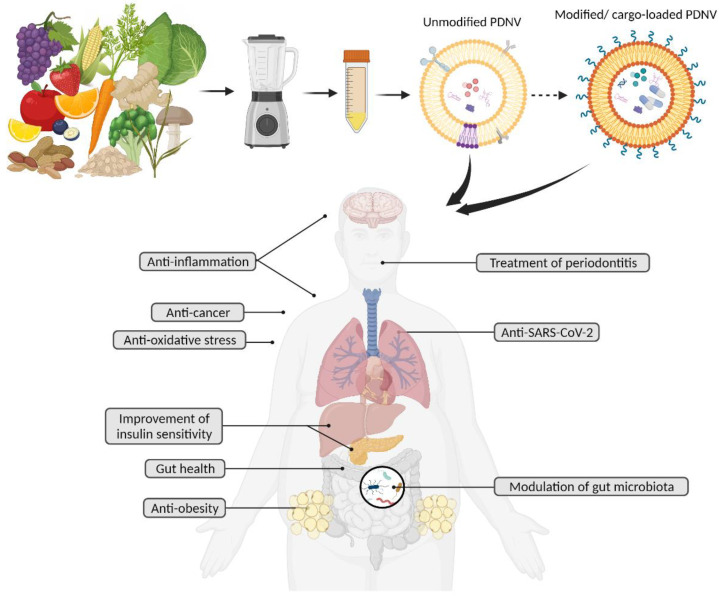
Unmodified PDNVs and modified PDNVs possess various potential health benefits.

PDNVs emerged as a novel class of bioactive food components with intrinsic health benefits (Figure 2). Studies summarized here showed that nanovesicles from various edible plants can serve as cross-species messengers. As the field expands, we anticipate revelations around what type of plant populations are responsible for specific biological activities and the determination of their primary targets. This could potentially be the groundwork for the personalized prescription of PDNVs. For example, when orally given to mice, grape-derived nanovesicles targeted intestinal stem cells [62,76], broccoli-derived nanovesicles targeted intestinal dendritic cells [50], grapefruit-derived nanovesicles were preferentially taken up by intestinal macrophages [51], and ginger-derived nanovesicles were taken up equally by intestinal macrophages and intestinal epithelial cells [34]. Another compelling aspect of PDNVs is that they can be developed as nanocarriers that efficiently deliver cargoes to the target location inside the human body since they are advantageous over artificial drug carriers due to their nano-size, low toxicity, safety, and potential for mass production. 

The development of PDNVs as marketable products needs to consider several factors. First, there is evidence that specific industrial processing removes PDNVs. For instance, compared with orange juice extracted using a juicer, orange juice with pulp (juice sacs) prepared by industrial processing has nanovesicles with altered morphology. On the other hand, orange juice from concentrates does not have nanovesicles [22]. Second, the integrity of PDNVs is subject to change with temperature, pH, and other processing conditions necessary to extend shelf life, but this may negatively impact the therapeutic potentials of the particles. For example, the destruction of stability by boiling or sonication removed citrus nanovesicles’ anti-proliferative effects [24]. In addition, studies on storage conditions for PDNVs are limited. Kim et al. tested the efficacy of preservatives on the stability of nanovesicles derived from *Dendropanax morbifera* leaf with varying temperatures at −20, 4, 25, and 45 °C by comparing physico-chemical properties (pH, size, protein content, and surface charge) and cellular uptake ability, but did not test whether the post-storage PDNVs preserve biological activity in recipient cells [111]. Similarly, another study showed that ginseng-derived nanovesicles retained stability after 60 days of storage post freeze-dry, but their bioactivity after the storage was not measured [112]. Third, there is a lack of consensus on isolation methods and implications are not clearly defined. For example, when different isolation methods (e.g., ultracentrifugation, PEG-based precipitation, and size-exclusion chromatography) were compared, different size distributions of the nanoparticles were observed with varying extents of heterogeneity [45]. The average sizes of nanovesicles were also different, yielding 148.2, 134.2, and 98.8 nm for PEG precipitation, ultracentrifugation, and size-exclusion chromatography, respectively [45]. Thus, a future study is warranted for the development of the technology that enables reproducible PDNV mass production and long shelf life. Last but not least, an investigation on the safety of PDNVs needs to be undertaken. Although no study has alerted or measured the presence of toxic chemicals such as herbicides and pesticides in the isolated PDNVs, given that mammalian exosomes release harmful or unwanted substances as a self-survival mechanism [113,114], PDNVs may contain a concentrated form of poisonous substances [115]. Therefore, rigorous analysis on the PDNVs’ cargo need to be carried out.

The application of PDNVs as a nanocarrier includes issues such as low drug loading capacity, and control of size distribution of carriers. Moreover, most of the intervention studies are based on cell and mouse models, which leaves questions unanswered as to their long-term effects and physiological ramifications. Currently, we found only one clinical study published the preliminary results [3]. Therefore, extensive pre-clinical and large-scale clinical research is warranted to ensure safety for commercial implementation. Despite these challenges, the field is worth investigating as a broad array of cellular communications via transfer of inherent or loaded cargoes can be accomplished with PDNVs. 

## Figures and Tables

**Table 1:** Main characterization methods of PDNVs.

Parameter	Methods	Features
Size	Dynamic light scattering (DLS)	Fast and easy; not suitable for heterogenous, poly-dispersed particle solution
	Nanoparticle tracking analysis (NTA)	Fast and easy; measures concentration; not suitable for poly-dispersed particle solution
	Flow cytometry	High throughput; not suitable for small particles
	Electron microscopy	High resolution; low throughput; particles not in native the state
	Atomic force microscopy (AFM)	Provides morphology; low throughput
	Tunable resistive pulse sensing (tRPS)	Highly accurate; measures concentration and charge; pores can be clogged by particles
Content	PCR	DNA/RNA
	Western Blot	Protein
	Mass spectrometry	Protein or lipid
	ELISA	Protein
	Sequencing	DNA/RNA
